# Prevalence of virulence factor, antibiotic resistance, and serotype genes of *Pasteurella multocida* strains isolated from pigs in Vietnam

**DOI:** 10.14202/vetworld.2020.896-904

**Published:** 2020-05-15

**Authors:** Hung Vu-Khac, T. T. Hang Trinh, T. T. Giang Nguyen, X. Truong Nguyen, Thi Thinh Nguyen

**Affiliations:** Department of Biotechnology, Institute of Veterinary Research and Development of Central Vietnam, Nha Trang City, Vietnam

**Keywords:** antibiotic resistance, capsule serotype, *Pasteurella multocida*, virulence factors

## Abstract

**Aim:**

The study was conducted to determine the prevalence and characterization of the *Pasteurella multocida* isolates from suspected pigs in Vietnam.

**Materials and Methods:**

A total of 83 *P. multocida* strains were isolated from lung samples and nasal swabs collected from pigs associated with pneumonia, progressive atrophic rhinitis, or reproductive and respiratory symptoms. Isolates were subjected to multiplex polymerase chain reaction (PCR) for capsular typing, detection of virulence-associated genes and antibiotic resistance genes by PCR. The antimicrobial sensitivity profiles of the isolates were tested by disk diffusion method.

**Results:**

All the isolates 83/83 (100%) were identified as *P. multocida* by PCR: serogroup A was obtained from 40/83 (48.19%), serogroup D was detected from 24/83 strains (28.91%), and serogroup B was found in 19/83 (22.35%) isolates. The presence of 14 virulence genes was reported including adhesins group (*ptfA* – 93.97%, *pfhA* – 93.97%, and *fimA* – 90.36%), iron acquisition (*exbB* – 100%, and *exbD* – 85.54%), hyaluronidase (*pmHAS* – 84.33%), and protectins (*ompA* – 56.62%, *ompH* 68.67%, and *oma87* – 100%). The dermonecrotoxin *toxA* had low prevalence (19.28%). The antimicrobial susceptibility testing revealed that cephalexin, cefotaxime, ceftriaxone, ofloxacin, pefloxacin, ciprofloxacin, and enrofloxacin were the drugs most likely active against *P. multocida* while amoxicillin and tetracycline were inactive. The usage of PCR revealed that 63/83 isolates were carrying at least one of the drug resistance genes.

**Conclusion:**

Unlike other parts of the word, serotype B was prevalent among Vietnamese porcine *P. multocida* strains. The high antibiotic resistance detected among these isolates gives us an alert about the current state of imprudent antibiotic usage in controlling the pathogenic bacteria.

## Introduction

*Pasteurella multocida* is a normal inhabitant of the respiratory tract of healthy animals [1]; however, it is an enigmatic pathogen known for being associated with a diversity of respiratory syndromes that can affect a range of host species [2]. In swine, it causes progressive atrophic rhinitis (PAR) and is thought to play an important role in pneumonia [3]. Based on the lipopolysaccharide antigens, the *P. multocida* strains are divided into five capsular serogroups (A, B, D, E, and F) and 16 somatic serotypes [4]. Serogroups A, B, and D have been identified in pigs [5,6]. Both toxigenic and non-toxigenic strains of serogroups A and D can cause pneumonic pasteurellosis, whereas isolates of serogroups B cause hemorrhagic septicemia in pigs [6].

The pathogenicity of *P. multocida* strains is associated with various virulence factors, which included dermonecrotic toxin, diverse adhesions, iron acquisition protein, and outer membrane proteins (OMP) [7]. Since the pathogenic behavior of *P. multocida* could be predicted by the virulence factors and the serogroups, evaluation of these virulence factors is important. Antibiotics have been used widely for the treatment of pasteurellosis in animals, their prolonged and indiscriminate use has led to onset of resistance among various strains [8-10], therefore, limiting therapeutic option [11]. Moreover, the antibiotic resistance in pathogenic bacteria from food-producing animals and environmental sources is recognized as a global problem for public health [12]. Several studies have proved that the imprudent usage of antibiotics increases the high risk for the selection of resistant bacteria and promotes the spread of resistance genes located on plasmids, integrons, and transposons [11,13]. Antibiotic resistance of *P. multocida* strains vary according to host origin, time of infection, geographic location, antibacterial pretreatment, and accessibility of the isolates to the resistance genes present in the gene pool [11]. Therefore, it was necessary to carry out a study of antibiotic genes in the pathogenic *P. multocida* strains.

The present study investigates the prevalence of virulence factor, antibiotic resistance, and capsular serotype genes of *P. multocida* isolated from pigs with symptoms of pasteurellosis in Vietnam.

## Materials and Methods

### Ethical approval

The approval from the Institutional Animal Ethics Committee to carry out this study was not required as no invasive technique was used.

### Bacterial strains and DNA extraction

Eighty-three porcine strains of *P. multocida* were investigated in this study. The isolates were kindly provided by Vietnam Regional Veterinary Centers and originated from widespread geographic locations within North and Central Vietnam over 7 years (from 2011 to 2018). The strains were recovered from lung samples or nasal swabs of 56 cases of porcine pneumonia and 18 cases of PAR or suspected PAR. Nine isolates were associated with porcine reproductive and respiratory symptoms. All strains were cultivated on tryptic soy yeast extract agar (TSA), supplemented with 5% rabbit blood, and incubated for 18 h at 37°C. The identification of strains was first based on the standard biochemical tests, including Gram staining, cultivation on MacConkey agar, catalase assay, oxidase assay, and sugar fermentation. The strains were then confirmed by *P. multocida* species-specific polymerase chain reaction (PM-PCR), as described by Townsend *et al*. [6].

The confirmed strains were cultivated statistically in BHI broth medium, at 37°C. An aliquot of cultivated medium after overnight incubation (1 ml) was subjected for DNA extraction using the commercial kit QIAamp DNA Mini (Qiagen).

### Capsular typing

The capsular types of the isolates were determined by multiplex capsule PCR typing with the capsule-specific primer pairs, including *capA*, *capB*, and *capE* reaction and *capD* and *capF* reaction [14]. The primer sequences used in the multiplex capsule PCR assays are listed in Table-1. Each 10 µl volume of multiplex PCR reaction contained each primer within the 5 primer sets at a concentration of 3.2 μM, 1× HotStarTaq^®^ Master Mix (Qiagen) and 1 µl total DNA of each isolate. The standard cycling procedure was used as following: An initial denaturation at 95°C for 5 min, followed by 30 cycles of denaturation at 95°C for 30 s, annealing at 55°C for 30 s, extension at 72°C for 30 s, and a final extension at 72°C for 5 min. The amplified products were separated by electrophoresis in 1% agarose gels and visualized by ethidium bromide staining.

**Table-1:** Primers used for the detection of virulence-associated genes, capsule serotypes in strains of *Pasteurella multocida.*

Gene	Function	Primer sequence	PCR product size (bp)	References
*pfhA*	Filamentous hemagglutinin	TTCAGAGGGATCAATCTTCG AACTCCAGT TGGTTTGTCG	286	[8]
*ptfA*	Adhesins	TGTGGAATTCAGCATTTTAGTGTGTC TCATGAATTCTTATGCGCAAAATCCTGCTGG	468	[8]
*fimA*	Adhesins	CCATCGGATCTAAACGACCTA AGTATTAGTTCCTGCGGGTG	866	[8]
*exbB*	Iron acquisition	TTGGCTTGTGATTGAACGC TGCAGGAATGGCGACTAA A	283	[8]
*exbD*	Iron acquisition	CGTTCTGATTACAGCCTCTT AACGAAATCTTGGAAACTGG	247	[8]
*pmHAS*	Hyaluronidase	TCAATGTTTGCGATAGTCCGTTAG TGGCGAATGATCGGTGATAGA	430	[8]
*ompA*	Protectins	CGCATAGCACTCAAGTTTCTCC CATAAACAGATTGACCGAAACG	201	[8]
*ompH*	Protectins	CGCGTATGAAGGTTTAGGT TTTAGATTGTGCGTAGTCAAC	438	[8]
*oma87*	Protectins	GGCAGCGAGCAACAGATAACG TGTTCGTCAAATGTCGGGTGA	838	[8]
*toxA*	Toxins	CTTAGATGAGCGACAAGG GAATGCCACACCTCTATAG	864	[19]
*KMT1*	Specific for *P. multocida*isolates	ATCCGCTATTTACCCAGTGG GCTGTAAACGAACTCGCCAC	457	[6]
*hyaD-hyaC*	Serogroup A *cap*	GATGCCAAAATCGCAGTCAG TGTTGCCATCATTGTCAGTG	1044	[14]
*bcbD*	Serogroup B *cap*	CATTTATCCAAGCTCCACC GCCCGAGAGTTTCAATCC	760	[14]
*dcbF*	Serogroup D *cap*	TTACAAAAGAAAGACTAGGAGCCC CATCTACCCACTCAACCATATCAG	657	[14]
*ecbJ*	Serogroup E *cap*	TCCGCAGAAAATTATTGACTC GCTTGCTGCTTGATTTTGTC	511	[14]
*fcbD*	Serogroup F *cap*	AATCGGAGAACGCAGAAATCAG TTCCGCCGTCAATTACTCTG	851	[14]

### Antibiotic susceptibility

All of *P. multocida* isolates were tested against 16 antimicrobial agents (Oxoid) by the disk diffusion method with the antimicrobial concentration as follows: Amikacin (30 µg), ampicillin (10 µg) amoxicillin (10 µg), cephalexin (30 µg), cefotaxime (30 µg), ceftriaxone (30), cephalothin (30 µg), erythromycin (15 µg), kanamycin (30 µg), gentamicin (10 µg), ofloxacin (5 µg), pefloxacin (5 µg), ciprofloxacin (5 µg), enrofloxacin (5 µg), chloramphenicol (30 µg), and tetracycline (30 µg). The tests were carried out according to the guideline of the Clinical and Laboratory Standards Institute [15]. The strains were grown in BHI media until getting OD_600nm_~0.5, and then were spread evenly on TSA agar plates supplemented with 5% rabbit blood. The plates were allowed to dry for a few minutes and the antimicrobial disks were placed on the surface of the plates. Then, they were incubated at 37°C for 18 h. The inhibition zones were measured with a ruler to the nearest millimeter. The strains were classified as sensitive, intermediate, and resistant, using the zone diameter standards provided by the CLSI [15].

### Detection of virulence and antibiotic resistance genes

The virulence genes and antibiotic resistance genes were tested by the PCR with specific primer pairs listed in Tables-1 and 2, respectively. The standard PCR mixture, the thermal cycling procedure and the result analysis step were performed similarly with the capsular typing PCR assays as described above. Except the tetO- detecting PCR assays, the extension time of the thermal cycling procedure was increase to 1 min and the assay results were analyzed on 0.8% agarose gel since the expected assay products were 1.8 kb. The appropriate positive and negative controls used in this study were clinical isolates of *P. multocida* that was confirmed in advance by both PCR and sequencing methods.

**Table-2:** Primer pairs and PCR procedure for the detection of antibiotic resistance genes.

Antibiotic group	Gene	Primer sequence	PCR product size (bp)	References
β-lactam	*bla*_TEM_	GAGTATTCAACATTTTCGT ACCAATGCTTAATCAGTGA	852	[32]
	*bla*_ROB1_	CATTAACGGCTTGTTCGC CTTGCTTTGCTGCATCTTC	856	[32]
Tetracycline	*tetB*	CCTTATCATGCCAGTCTTGC ACTGCCGTTTTTTTCGCC	774	[32]
	*tetH*	ATACTGCTGATCACCGT TCCCAATAAGCGACGCT	1076	[32]
	*tetO*	TAACTTAGGCATTCTGGCTC TCAAGCAGACTCCCTGCCCATTTGT	1801	[32]
Chloramphenicol	*floR*	CACGTTGAGCCTCTATATGG ATGCAGAAGTAGAACGCGAC	885	[35]
Gentamicin	*aacA4*	CTCGAATGCCTGGCGTGTTTTTGCGATGCTCTATGAGTGGCTA	482	[34]

## Results

### Distribution of capsule biosynthesis and virulence-associated genes in *P. multocida* isolates

**Figure-1:**
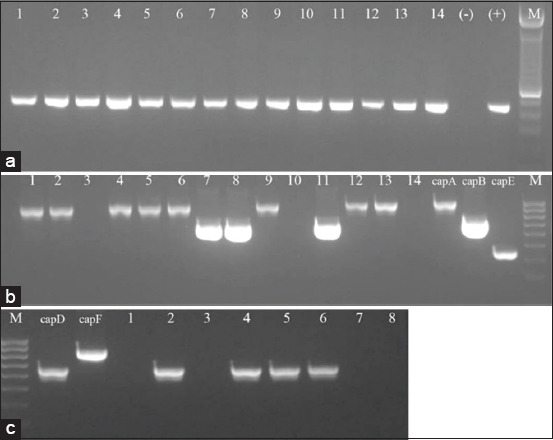
Species-specific polymerase chain reaction (PCR) and multiplex PCR for capsular typing of *Pasteurella multocida*. (a) *P. multocida confirmation by species-specific PCR. 1-11*: Samples; (−): Negative control; (+): Positive control (457 bp); M: 100 bp DNA ladder. (b) Multiplex reactions of *capA*, *capB*, *capE*. 1-14: samples; capA: *capA* positive control (1044 bp); capB: *capB* positive control (760 bp); capE: *capE* positive control (511 bp); M: 100 bp DNA ladder. (c) Multiplex reactions of *capD* and *capF*. M: 100 bp DNA ladder; capD: *capD* positive control (657bp); capF: *capF* positive (851 bp), 1-8: Samples.

The biochemical tests determined that all 83 isolates provided by Vietnam Regional Veterinary Centers showed *P. multocida*-specific biochemical characteristics, including Gram-negative coccobacilli, not growing on MacConkey agar, non-hemolytic, positive for catalase, oxidase enzymes, and glucose, galactose, fructose, and sucrose but not lactose fermentation. According to these results, PCR assays with the species-specific primers also confirmed that these 83 collected strains were exactly *P. multocida* (Figure-1a). Among these strains, the *capA*, *capD*, and *capB* genes were obtained from 40 (48.19%), 24 (28.91%), and 19 isolates (22.35%), respectively. However, neither of the isolates harbored *capE* and *capF* genes (Table-3 and Figure-1b and c).

**Table-3:** The distribution of capsule biosynthesis and virulence-associated genes of *Pasteurella multocida* detected by PCR.

Process or enzyme	Gene	Absolute and relative frequency (%) (n=83)
Capsule biosynthesis genes	*hyaD-hyaC (capA)*	40 (48.19)
	*bcbD (capB)*	19 (22.35)
	*dcbF (capD))*	24 (28.91)
	*ecbJ (capE)*	0 (0)
	*fcbD (capF)*	0 (0)
Virulence-associated genes	*fimA*	75 (90.36)
	*pfhA*	78 (93.97)
	*ptfA*	78 (93.97)
Iron acquisition genes	*exbB*	83 (100)
	*exbD*	71 (85.54)
Outer membrane protein	*ompA*	47 (56.62)
	*ompH*	57 (68.67)
	*Oma87*	83 (100)
Hyaluronic acid synthetase	*pmHAS*	70 (84.33)
Dermonecrotic toxin	*toxA*	16 (19.28)

PCR=Polymerase chain reaction

**Figure-2:**
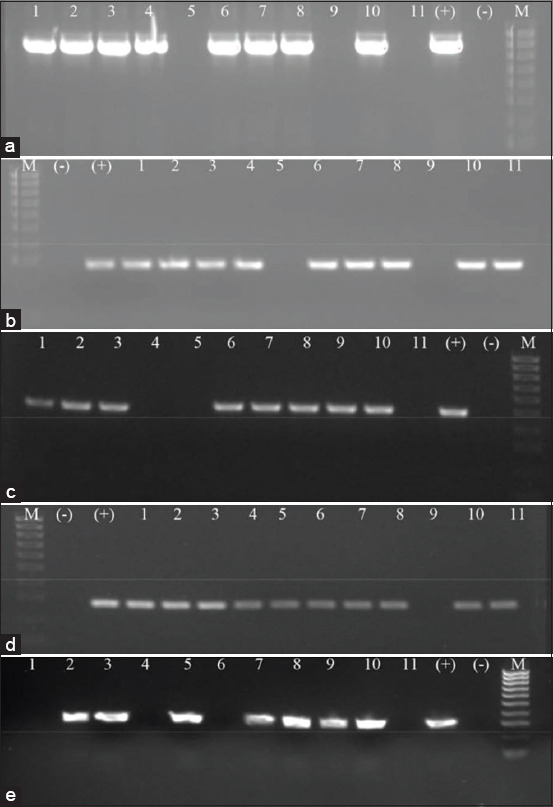
Polymerase chain reaction assay checking the prevalence of *fimA* (a), *pfhA* (b), *ptfA* (c), *exbB* (d), and *exbD* (e) genes in *Pasteurella multocida* isolates. M: 100 bp DNA ladder; (−): Negative control; (+): Positive control 1-11: Samples.

**Figure-3:**
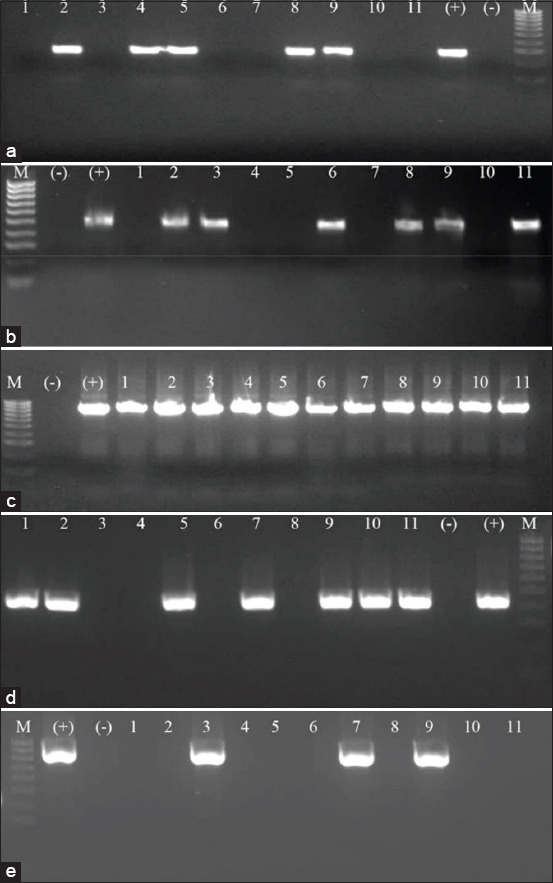
Polymerase chain reaction assay checking the prevalence of *ompH* (a), *ompA* (b), *Oma87* (c), *pmHAS* (d), *toxA* (e) genes in *Pasteurella multocida* isolates. M: 100 bp DNA ladder; (−): Negative control; (+): Positive control 1-11: Samples.

The occurrence of ten virulence-associated genes of *P. multocida* isolates was determined by PCR (Table-3). The results showed that more than 90% of isolates carried the genes *fimA* (90.36%), *pfhA* (93.97%), and *ptfA* (93.97%) (Figure-2a-c). Among the iron acquisition genes studies, the *exbB* gene was identified in 100% of isolates, but 85.54% of the isolates carried the *exbD* gen (Figure-2d and e). The genes encoding for outer membrane proteins including *ompA*, *ompH*, and *oma87* were detected in 56.62%, 68.67%, and 100%, respectively (Figure-3a-c). The *pmHAS* gene was observed in 84.33% of isolates (Figure-3d). Dermonecrotoxin gene (*toxA*) was found only in 19% of the isolates (Figure-3e). Some virulence genes exhibited distinctive associations with serogroups A and D. The genes *toxA* and *FimA* are positively associated with serogroup D and the *pmHAS* with serogroup A. The *exbD* gene and *oma87* were found in all three serogroups A, B, and D.

### Antibiotic susceptibility

Eighty-three strains of *P. multocida* isolated from clinically sick pigs were tested for resistance to 16 antibiotics (Table-4). Most of the prevalent phenotypes observed were resistance to amoxicillin (75.9%) and tetracycline (59%), followed by kanamycin (15.7%), amikacin (15.7%), gentamicin (14.5%), ampicillin (9.6%), and erythromycin (9.6%). Less than 5% of the strains were resistant to chloramphenicol (4.8%). No resistance to cephalexin, cefotaxime, ceftriaxone, ofloxacin, pefloxacin, ciprofloxacin, and enrofloxacin could be found.

**Table-4:** Antimicrobial susceptibility pattern of 83 *Pasteurella multocida* isolates tested with the disk diffusion method.

No.	Antimicrobial agent	Sensitivity of isolates to antimicrobial agents		

		Sensitive (%)	Intermediate (%)	Resistant (%)
1	Amoxicillin	13 (15.7)	7 (8.4)	63 (75.9)
2	Tetracycline	24 (28.9)	10 (12)	49 (59)
3	Kanamycin	70 (84.3)	-	13 (15.7)
4	Amikacin	68 (81.9)	2 (2.4)	13 (15.7)
5	Gentamicin	71 (85.5)	-	12 (14.5)
6	Ampicillin	70 (84.3)	5 (6)	8 (9.6)
7	Erythromycin	66 (79.5)	9 (10.8)	8 (9.6)
9	Chloramphenicol	79 (95.2)	-	4 (4.8)
10	Cephalexin	83 (100)	-	0 (0)
11	Cefotaxime	83 (100)	-	0 (0)
12	Ceftriaxone	79 (95.2)	4 (4.8)	0 (0)
13	Ofloxacin	83 (100)	-	0 (0)
14	Pefloxacin	83 (100)	-	0 (0)
15	Ciprofloxacin	81 (97.6)	2 (2.4)	0 (0)
16	Enrofloxacin	83 (100)	-	0 (0)

### Occurrence of antibiotic resistance genes

**Figure-4:**
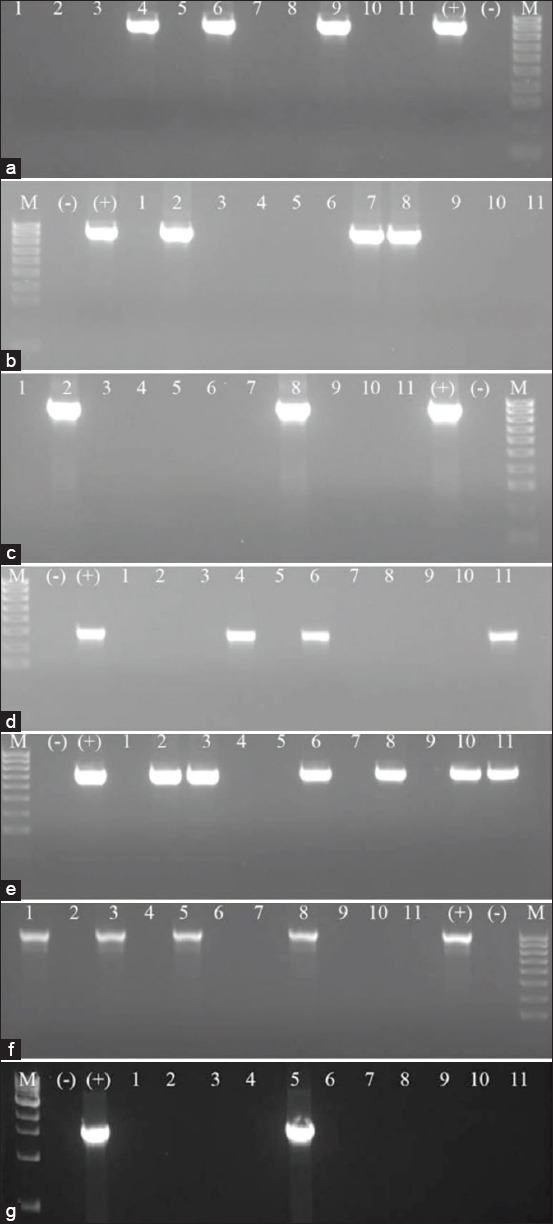
Polymerase chain reaction assay checking the prevalence of *bla*_TEM_ (a), *bla*_ROB1_ (b), *floR* (c), *aacA4* (d), *tetH* (e), *tetB* (f), *tetO* (g) genes in *Pasteurella multocida* isolates. M: 100 bp DNA ladder (a-f) and 1kb DNA ladder (g); (−): Negative control; (+): Positive control; 1-11: Samples.

The prevalence of the resistance genes for ampicillin, gentamycin, chloramphenicol, and tetracycline is shown in Table-5. The resistance to ampicillin was mediated by *bla*_TEM_ or *bla*_ROB1_ genes; among 83 strains tested, 15 were positive for *bla*_TEM_ and/or *bla*_ROB1_ genes (Figure-4a and b). The correlation between genotype (absence or presence of resistance gene) and phenotype (susceptibility) was poor for ampicillin (4/8 resistant strains carried *bla*_TEM_ and/or *bla*_ROB1_ genes).

**Table-5:** The distribution of major resistance genes for ampicillin, chloramphenicol, gentamicin, tetracycline, and correlation between genotype and phenotype.

Antimicrobial agent	Resistance genes	Number of positive isolates (%)	Correlation between genotype and phenotype
Ampicillin (n=83)	*bla*_TEM_	6 (7.2)	
	*bla*_ROB1_	12 (14.5)	
	At least one	15 (18)	4/8 (50)
Chloramphenicol (n=83)	*floR*	5 (6)	1/4 (25)
Gentamicin (n=83)	*aacA4*	7 (8.4)	7/12 (58)
Tetracycline (n=83)	*tetB*	38 (45.8)	
	*tetH*	18 (21.7)	
	*tetO*	4 (4.8)	
	At least one	52 (62.65)	39/49 (79.6)

Florfenicol is a fluorinated derivative of chloramphenicol. The florfenicol resistance gene, *floR*, confers resistance to chloramphenicol and florfenicol. In this study, 5/83 strains were positive for *floR* gene (Figure-4c), in which, only 1/4 chloramphenicol resistant strains harbored this gene. In contrast, the correlation between genotype and phenotype for gentamicin was good as 7/12 susceptible strains carried the *aacA4* gene (Figure-4d).

The resistance to tetracycline of *P. multocida* was documented to be mediated by *tetB*, *tetH*, and *tetO* genes [16]. In this study, we revealed all of these genes appearing among 83 isolated strains. There were totally 52/83 isolates carrying at least one of these resistance genes, in which, the *tetB* was the most prevalent (38/52), followed by the *tetH* gene (18/52) and *tetO* gene (4/52) (Figure-4e-g). The agreement between genotype and phenotype for tetracycline was high as 39/49 tetracycline-resistant trains have at least one resistance gene.

## Discussion

This study is the first report of the prevalence of virulence factor, antibiotic resistance, and capsular virulence genes of *P. multocida* strains isolated from pigs in Vietnam. Pasteurellosis is common disease of pigs worldwide with specific serotype and pathotype associated with the respiratory disease [17]. However, the distribution and prevalence of serotype and pathotype can vary considerably from region to region and over the time in a given region [8,18,19]. The present study results in the detection of a higher percentage of serogroup A (48.19%) than serogroup D (28.91%). Similar results were reported in China (49.3% vs. 47.6%) [19], India (66.66% vs. 33.33%) [20], and in England and Wales (74% vs. 22%) [5]. In contrast, in Germany [7] and Malaysia [21], the prevalence of strains of the serogroup D is higher than that of serogroup A. The serogroup B was none or less commonly found in *Pasteurella multocida* isolated from pigs in England and Wales [5], Germany [22], and China [8,19] and this serogroup is associated with strains isolated from cattle to buffalos [7]. However, in this study, we found 22.35% of 83 isolates belong to serogroup B. In Vietnam, animal husbandry practices such as cohabitation of various species of animal including cattle, buffalo, and use of common grazing ground may lead to spread of infection among all such host species. Moreover, in a previous study Townsend *et al*. [6], have shown the occurrence of acute septicemic pasteurellosis in pigs due to hemorrhagic septicemia associated type B genotypically identical to those that cause hemorrhagic septicemia in cattle and buffalo in south of Vietnam.

Although the majority of virulence genes presented a similar distribution between serogroups, some were significantly associated with a specific capsular type, such as *pmHAS* gene with serogroup A. In this study, we found that all the isolates belong to serogroup A carried the *pmHAS* gene whereas only 70.83% of the isolates of serogroup D and 68.42% of the isolates of serogroup B harbored this gene. Our results are in agreement with those of the previous studies [1,8], which showed a positive association of *pmHAS* gene to serogroup A but not serogroup D. It is well documented that the *toxA* gene, encoding for a dermonecrotic toxin (PMT-*P. multocida* toxin), presented a significant association to serogroup D. This toxin induces osteolysis in the turbinate bones, a process playing an important role in the PAR [5]. In the present study, we found that a total of 16 isolates belonged to serogroup D carried the *toxA* gene, but we did not find this gene (*toxA*) in isolates of serogroups A and B. Similarly, the positive association of the *toxA* gene with serogroup D among *P. multocida* isolated from swine has been reported in Brazil [1], China [8], and Germany [7].

The basic prerequisite for bacterial infection is its attachment to host cell and therefore, adhesion is considered to be one of potential virulence factors [23]. In this study, the high prevalence of a number of adhesion-related genes such as *fimA, pfhA*, and *ptfA* was observed indicating that either proteins act synergistically or are required at different stages of colonization/infection. Filamentous hemagglutinins encoded by *pfhA* gene play an important role in the initial colonization of the upper respiratory tract and the frequency of this gene varies greatly among strains of *P. multocida*. This gene has been documented to be an important epidemiological marker and associated with the occurrence of disease in cattle, swine, and sheep [7,24,25]. Interestingly, we found a very high prevalence of *pfhA* (93.97%) gene irrespective of its capsular type. Similar findings have also been reported by the previous studies [1,23,26].

The ability of the pathogens to obtain iron is a key feature during the infection process [27]. The high prevalence of two iron acquisition protein (*exbB and exbD*) has been reported in *P. multocida* strains isolated from pigs in China [8] and Brazil [1]. The similar results for *exbB and exbD* genes were observed in the present study. We reported *exbB* gene in 100% of isolates, and *exbD* gene in 85.22% of the isolates. In agreement with the previous study [7], *ompA*, *ompH*, and *oma87* genes, which encoded porins of *P. multocida*, were detected, respectively, in 56.62%, 68.67%, and 100% of isolates in the current study. These findings suggest that OMPs have a significant role in the host-pathogen interaction.

Antimicrobial therapy is still the most effective tool for the treatment of infectious diseases caused by *P. multocida*, as the strains were susceptible to the majority of antibiotics commercially available. In this study, we found that the most active drugs against isolated *P. multocida* strains were cephalexin, cefotaxime, ceftriaxone, ofloxacin, pefloxacin, ciprofloxacin, and enrofloxacin. These antibiotics were also reported to be susceptible to *P. multocida* strains in France [28] and China [8]. The prevalence of resistance to conventional antibiotics, including kanamycin, amikacin, gentamicin, ampicillin, and erythromycin was found to be in 9.6-15.7% of 83 *P. multocida* trains. Therefore, using these drugs for preventive and treatment of *P. multocida* infection may not be effective. The high number of *P. multocida* strains resistant to amoxicillin and tetracycline has been reported in Cuba [29], China [8], and in Taiwan (amoxicillin) [30], which is similar to our results, as we found 75.9% and 59% of 83 strains resistant to amoxicillin and tetracycline, respectively. In contrast, in Brazil, the high number of *P. multocida* strains was documented to be susceptible to amoxicillin and tetracycline [1]. This may explain that the prevalence of antibiotic resistance of *P. multocida* strains varies according to geographical origin and antimicrobial treatments previously applied in the given population. Chloramphenicol has been known to produce major adverse effects in human, and it is only applied as final solution when safe antibiotics cannot be used. In this study, we found only 4.8% of strains resistant to this drug. Similar results were reported in Spain [31].

In parallel with the phenotypic antibiotic resistance testing, we also investigated the prevalence of resistance genes for ampicillin, gentamicin, chloramphenicol, and tetracycline. The results showed that 63/83 strains carried at least one resistance gene tested indicating that these genes have a major role in conferring resistance among the strains investigated. Moreover, the presence of different genes within the same strain, encoding resistance to the same antibiotic agent, was detected in eight strains. We have found that such strains are more frequently associated with multiresistance than expected by chance. The finding of *tetB*, *tetH*, and *tetO* genes in 52 strains provided a likely explanation for phenotypic resistance of those strains to tetracycline as 39/49 *P. multocida* strains resistant to tetracycline have at least one resistance gene, in which the *tetB* gene is the most prevalent. Similar to our finding, the strong correlation between genotype and phenotype for tetracycline has been reported in *P. multocida* strains in Australia [32] and Spain [31]. *aacA4* is an aminoglycoside-resistant genes and is suggested for gentamicin resistance [33]. This gene was revealed to be present frequently in *P. multocida* strains isolated in China [34]. However, our results show that *aacA4* is less frequent among *P. multocida* strains as only 7/83 strains positive with it (Table-5). The *floR* gene was firstly detected in 1996 on a plasmid in the fish pathogen *Photobacterium damselae* subsp. *Piscicida* [35]. In this study, we found five *P. multocida* strains harboring this gene and only one strain showing phenotypic chloramphenicol resistance. The resistance to β-lactam antibiotics in Gram-negative bacteria is mediated primarily by β-lactamases. In this study, we found six and 12 out of 83 *P. multocida* strains harboring *bla*_TEM_ and *bla*_ROB1_ genes, respectively. Even though the previous study documented that ampicillin and penicillin resistance have been linked to *bla*_TEM_ gene in Gram-negative bacteria [36], our results suggest that *bla*_ROB1_ is major player for β-lactam antibiotic resistance in *P. multocida*.

## Conclusion

The present study provides epidemiological information on the diversity and virulence gene properties of Vietnamese porcine *P. multocida* strains in comparison to its equivalents in other countries. The result showed that unlike other parts of the word the serotype B was prevalent among Vietnamese porcine *P. multocida* strains. This study has also identified a number of resistance genes associated with most of phenotypic resistance observed in these strains. The information about virulence and antibiotic resistance genes suggests that more attention should be paid to the prudent use of antibiotics and vaccination should be our favored solution to prevent the negative evolving state of antibiotic resistance.

## Author’s contributions

TTHT and TTGN directly carried out the experiments. HV conceived the original idea, supervised the project, and prepared the manuscript with the support from TTGN, XTN and TTN. All authors have read and approved the final manuscript.
